# Incremental Validity of Character Strengths as Predictors of Job Performance Beyond General Mental Ability and the Big Five

**DOI:** 10.3389/fpsyg.2021.518369

**Published:** 2021-03-12

**Authors:** Claudia Harzer, Natalia Bezuglova, Marco Weber

**Affiliations:** ^1^Department of Psychology, Technical University Darmstadt, Darmstadt, Germany; ^2^Department of Psychology, University of Greifswald, Greifswald, Germany

**Keywords:** character strengths, job performance, general mental ability, Big Five, incremental validity

## Abstract

Over the last decades, various predictors have proven relevant for job performance [e.g., general mental ability (GMA), broad personality traits, such as the Big Five]. However, prediction of job performance is far from perfect, and further potentially relevant predictors need to be investigated. Narrower personality traits, such as individuals' character strengths, have emerged as meaningfully related to different aspects of job performance. However, it is still unclear whether character strengths can explain additional variance in job performance over and above already known powerful predictors. Consequently, the present study aimed at (1) examining the incremental validity of character strengths as predictors of job performance beyond GMA and/or the Big Five traits and (2) identifying the most important predictors of job performance out of the 24 character strengths, GMA, and the Big Five. Job performance was operationalized with multidimensional measures of both productive and counterproductive work behavior. A sample of 169 employees from different occupations completed web-based self-assessments on character strengths, GMA, and the Big Five. Additionally, the employees' supervisors provided web-based ratings of their job performance. Results showed that character strengths incrementally predicted job performance beyond GMA, the Big Five, or GMA plus the Big Five; explained variance increased up to 54.8, 43.1, and 38.4%, respectively, depending on the dimension of job performance. Exploratory relative weight analyses revealed that for each of the dimensions of job performance, at least one character strength explained a numerically higher amount of variance than GMA and the Big Five, except for individual task proactivity, where GMA exhibited the numerically highest amount of explained variance. The present study shows that character strengths are relevant predictors of job performance in addition to GMA and other conceptualizations of personality (i.e., the Big Five). This also highlights the role of socio-emotional skills, such as character strengths, for the understanding of performance outcomes above and beyond cognitive ability.

## Introduction

Job performance is seen as a decisive production resource, especially in industrial societies. Therefore, among the core goals of personnel selection is to hire applicants who will perform well in the future. Over the last 30 years, researchers have investigated various variables in order to identify relevant predictors of job performance. These potential predictors include (but are not limited to) broad *personality traits* (e.g., Tett et al., 1991; Barrick et al., 2001; Salgado, 2003), *general mental ability* (Schmidt and Hunter, 1998; Salgado and Anderson, 2003; Hülsheger et al., 2007; e.g., Kramer, 2009; GMA), as well as narrow traits, such as *self-esteem* (e.g., Judge and Bono, 2001; Sekiguchi et al., 2008), facets of conscientiousness (e.g., Dudley et al., 2006), or assertiveness as facet of extraversion (e.g., Bergner et al., 2010). However, prediction of job performance is far from perfect, and further potentially relevant predictors need to be investigated to further improve it.

As a result of the positive psychology movement, perspectives and constructs that were long neglected in psychological research (e.g., Seligman and Csikszentmihalyi, 2000) are increasingly taking center stage. Among these is the concept of “character strengths” (e.g., Peterson and Seligman, 2004), which represents a positive perspective on personality traits as opposed to more neutral (e.g., the Big Five traits, such as extraversion or conscientiousness; Ostendorf, 1990) or negative ones (e.g., the Dark Triad of narcissism, psychopathy, and Machiavellianism; Paulhus and Williams, 2002). These character strengths may be useful additional predictors of job performance and, therefore, are the center of attention in the present paper.

### Character Strengths

According to Peterson and Seligman (2004), character strengths are individual differences that are positively valued across cultures and find expression in individuals' thoughts (e.g., considering the consequences of one's behavior before acting), feelings (e.g., enjoying teamwork), and behaviors (e.g., engaging in learning activities). Character strengths are narrow, trait-like personality characteristics; they exhibit a reasonable amount of stability over time and situations, but are nevertheless influenced by life circumstances and might therefore change over the life course or as the result of training (Peterson and Seligman, 2004; see also Gander et al., 2021). Peterson and Seligman (2004) identified 24 character strengths through intensive research employing numerous historical, philosophical, and psychological sources, with the aim of more systematically describing personality from a positive perspective. These character strengths are distinct from one another and measurable. Table 1 presents the 24 character strengths included in the Values in Action classification of strengths (Peterson and Seligman, 2004) as well as short descriptions defining them.

**Table 1:** The 24 character strengths included in the Values in Action classification of strengths (Peterson and Seligman, 2004) and short descriptions defining the strengths.

**1. Cognitive strengths that entail the acquisition and use of knowledge**
*Creativity [originality, ingenuity]*: thinking of novel and productive ways to conceptualize and do things; includes but is not limited to artistic achievement *Curiosity [interest, novelty-seeking, openness to experience]*: taking an interest in all of ongoing experience for its own sake; finding subjects and topics fascinating; exploring and discovering *Judgment [open-mindedness, critical thinking]*: thinking things through and examining them from all sides; not jumping to conclusions; being able to change one's mind in light of evidence; weighing all evidence fairly *Love of learning*: mastering new skills, topics, and bodies of knowledge, whether on one's own or through formal instruction; related to curiosity but goes beyond it to describe the tendency to systematically add to what one knows *Perspective [wisdom]*: being able to provide wise counsel to others; having ways of looking at the world that make sense to oneself and to others
**2. Emotional strengths that involve the exercise of will to accomplish goals in the face of opposition, external or internal**
*Bravery [valor]*: *not* shrinking from threat, challenges, difficulty, or pain; speaking up for what is right even in the face of opposition; acting on one's convictions even if unpopular; includes but is not limited to physical bravery *Perseverance [persistence, industriousness]*: finishing what one starts; persisting in a course of action in spite of obstacles; “getting it out the door”; taking pleasure in completing tasks *Honesty [authenticity, integrity]*: speaking the truth but also more broadly presenting oneself and acting in a genuine and sincere way; being without pretense; taking responsibility for one's feelings and actions *Zest [vitality, enthusiasm, vigor, energy]*: approaching life with excitement and energy; not doing things halfway or halfheartedly; living life as an adventure; feeling alive and activated
**3. Interpersonal strengths that involve “tending and befriending” others**
*Capacity to love and be loved [short name: love]*: valuing close relations with others, in particular those in which sharing and caring are reciprocated; being close to people *Kindness [generosity, nurturing, care, compassion, altruistic love, “niceness”]*: doing favors and good deeds for others; helping them; taking care of them *Social intelligence [emotional intelligence, personal intelligence]*: being aware of the motives and feelings of other people and oneself; knowing what to do to fit into different social situations; knowing what makes other people tick
**4. Civic strengths that underlie healthy community life**
*Teamwork [citizenship, social responsibility, loyalty]*: working well as a member of a group or team; being loyal to the group; doing one's share *Fairness*: treating all people the same according to notions of fairness and justice; not letting personal feelings bias one's decisions about others; giving everyone a fair chance *Leadership*: encouraging a group of which one is a member to get things done and at the same time maintain good relations within the group; organizing group activities and seeing that they happen
**5. Strengths that protect against excess**
*Forgiveness [mercy]*: forgiving those who have done wrong; accepting the shortcomings of others; giving people a second chance; not being vengeful *Modesty [humility]*: letting one's accomplishments speak for themselves; not regarding oneself as more special than one is *Prudence*: being careful about one's choices; not taking undue risks; *not* saying or doing things that one might later regret *Self-regulation [self-control]*: regulating what one feels and does; being disciplined; controlling one's appetites and emotions
**6. Transcendental strengths that forge connections to the larger universe and provide meaning**
*Appreciation of beauty and excellence [awe, wonder, elevation; short name: appreciation]*: noticing and appreciating beauty, excellence, and/or skilled performance in various domains of life, from nature to art to mathematics to science to everyday experience *Gratitude*: being aware of and thankful for the good things that happen; taking time to express thanks *Hope [optimism, future-mindedness, future orientation]*: expecting the best in the future and working to achieve it; believing that a good future is something that can be brought about *Humor [playfulness]*: liking to laugh and tease; bringing smiles to other people; seeing the light side of life; making (not necessarily telling) jokes *Spirituality [religiousness, faith, purpose]*: having coherent beliefs about the higher purpose and meaning of the universe; knowing where one fits within the larger scheme of things; having beliefs about the meaning of life that shape one's conduct and provide comfort

*The character strengths are grouped together theoretically based on their content. The labels and expressions in brackets emphasize the family resemblance among the concepts to acknowledge the heterogeneity of strengths and minimize subtle (political or otherwise) connotations (Peterson and Seligman, 2004)*.

The character strengths are clustered into six groups (see Table 1). This was done on theoretical grounds rather than empirically (e.g., by factorial analyses) (Peterson and Seligman, 2004). By definition, character strengths contribute to individuals' fulfillment, flourishing, and thriving (Peterson and Seligman, 2004). Accordingly, research has shown meaningful relations between specific character strengths and favorable outcomes in different areas of life, including *physical health* (e.g., Proyer et al., 2017), *life satisfaction* (e.g., Park et al., 2004; Buschor et al., 2013), *psychological well-being* (e.g., Harzer, 2016), *school achievement* (e.g., Weber, 2018), and *vocational orientation among young people* (e.g., Proyer et al., 2012).

Several studies have highlighted the role of character strengths in the work context. The results stem from samples around the globe (e.g., Canada, Germany, Israel, Pakistan, Switzerland, and the US). For example, character strengths are related to work-related well-being. Specifically, higher scores on character strengths were associated with higher scores on beneficial outcomes, such as positive affect, work engagement, sense of meaning, job satisfaction, and lower stress (Peterson et al., 2010; Harzer and Ruch, 2015; e.g., Harzer et al., 2017; Heintz and Ruch, 2021). Another crucial work-related outcome is job performance.

### Job Performance

Job performance is a multi-faceted construct, as employees exhibit different performance-related behaviors at different times depending on the situation (e.g., Williams and Anderson, 1991; Borman et al., 1995; Coleman and Borman, 2000; Motowidlo, 2000; Viswesvaran and Ones, 2000; Griffin et al., 2007). Therefore, several dimensions of job performance have been considered in research.

**Figure 1:**
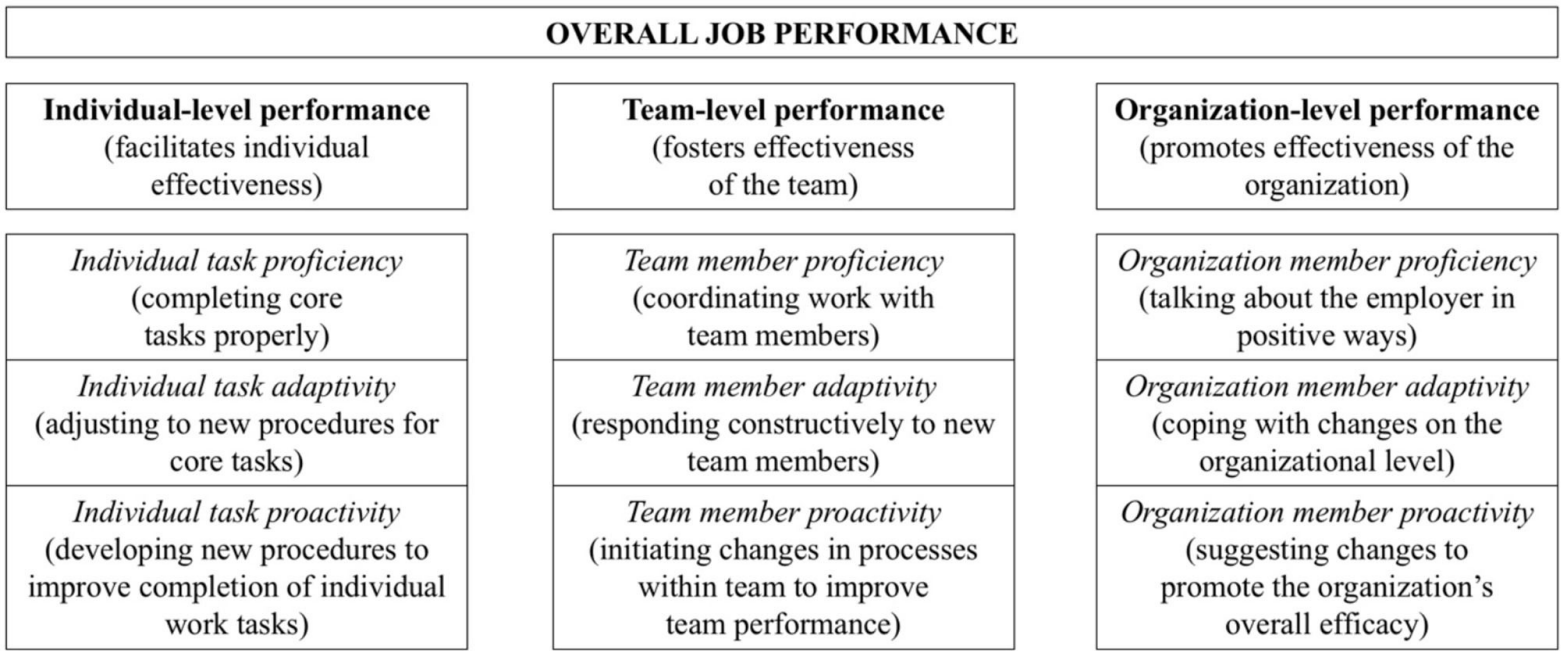
Components and brief definitions of Griffin's (2007) model of job performance.

Firstly, there are aspects of job performance that positively influence organizational effectiveness (e.g., Viswesvaran and Ones, 2000). These are in-role behavior (also known as task performance; e.g., Williams and Anderson, 1991) and extra-role behavior (also known as contextual performance or organizational citizenship behavior; e.g., Motowidlo, 2000). The latter includes aspects, such as job dedication (work motivation), interpersonal facilitation (support of co-workers), and organizational support (loyalty) (e.g., Coleman and Borman, 2000). In their model of positive work role performance, Griffin et al. (2007) offered a more fine-grained perspective on *productive work behavior* by distinguishing between proficiency of work-related behavior, adaptivity to change, and proactivity to improve processes on the individual, team, and organizational levels. Proficiency refers to the fulfillment of prescribed or predictable requirements of one's work role; adaptivity is related to coping with, reacting to, and supporting change; proactivity means initiating change in a self-started and future-directed way (Griffin et al., 2007). Figure 1 provides an overview of all components included in the model by Griffin et al. (2007) as well as brief definitions of the components in order to define productive work behavior as examined in the present paper.

Secondly, there are dimensions of job performance that negatively influence organizational effectiveness (e.g., Viswesvaran and Ones, 2000). These are termed *counterproductive work behavior* (also known as deviant behavior; e.g., Bennett and Robinson, 2000; Marcus and Schuler, 2004). Counterproductive work behavior or deviance at work “violates significant organizational norms and, in so doing, threatens the well-being of the organization or its members, or both” (Bennett and Robinson, 2000, p. 349). This behavior can be directed at the organization itself (organizational deviance; e.g., taking property from work without permission) or at organizational members (interpersonal deviance; e.g., making fun of someone at work) (Bennett and Robinson, 2000).

According to a number of meta-analyses (e.g., Schmidt and Hunter, 1998; Salgado and Anderson, 2003; Salgado et al., 2003; Hülsheger et al., 2007; Salgado and Moscoso, 2019) utilizing different data from different cultures and countries, GMA is a robust predictor of task performance (comparable with individual task proficiency) and overall productive work behavior (often termed overall job performance in the literature). The correlation between GMA and overall job performance is around 0.50. Research on the relationships between GMA and the other dimensions of productive and counterproductive work behavior is relatively scarce. However, Gonzalez-Mulé et al. (2014) in a meta-analysis showed that GMA is significantly positively related to organizational citizenship behavior (comparable with team-level and organization-level performance; correlation around 0.20) as well as negatively related to organizational deviance (correlation around −0.20). There was no systematic relationship between GMA and interpersonal deviance.

Due to the low incremental validity of other predictors beyond GMA, GMA has often been considered the best predictor of task performance and overall job performance (e.g., Schmidt and Hunter, 1998). Nevertheless, a number of meta-analyses (e.g., Barrick and Mount, 1991; Salgado, 1997, 2002; Hurtz and Donovan, 2000) have shown that personality traits, such as the Big Five, are potent predictors of job performance as well. For example, conscientiousness was the best predictor of overall job performance, task performance, team-level performance, and counterproductive work behavior among the Big Five across different occupations (correlations around 0.20). However, especially when focusing on specific occupations (e.g., customer service) and dimensions of job performance (e.g., counterproductive work behavior), the remaining Big Five dimensions were significant predictors as well.

### Character Strengths and Job Performance

Several studies have investigated the relations between character strengths and various dimensions of job performance, such as *individual-level performance* and its subdimensions (e.g., Cosentino and Castro Solano, 2012; Harzer and Ruch, 2014; Littman-Ovadia and Lavy, 2016; Harzer et al., 2017), *team-level and organization-level performance* and their subdimensions (e.g., Harzer and Ruch, 2014; Harzer et al., 2017; Littman-Ovadia and Raas-Rothschild, 2018), as well as *counterproductive work behavior* and its subdimensions (e.g., Littman-Ovadia and Lavy, 2016; Harzer et al., 2017). Research has repeatedly shown that character strengths are systematically correlated with various dimensions of job performance. For example, perseverance and honesty were positively related to individual-level performance; teamwork and fairness were positively related to team-level performance; and forgiveness and fairness were negatively related to counterproductive work behavior. This is in line with the definition of character strengths as personality traits that contribute to individuals' successes and performances in life (Peterson and Seligman, 2004).

However, the question arises as to what extent character strengths exhibit incremental validity as predictors of job performance beyond common predictors utilized in industrial and organizational psychological research and practice. The incremental validity of character strengths beyond GMA is of interest, as GMA is often considered the best predictor of job performance (e.g., Schmidt and Hunter, 1998). Therefore, examining whether or not other potential predictors of job performance significantly improve the prediction of job performance beyond GMA is of particular interest. Character strengths and GMA are two distinct psychological constructs that show by definition no substantial overlap (e.g., Peterson and Seligman, 2004), which implies that such personality characteristics may be very potential candidates explaining variance in job performance beyond GMA. However, to the best of our knowledge, no empirical evidence on the relations between character strengths and GMA is available so far. Nevertheless, as character strengths show substantial relations with various dimensions of job performance and are theoretically distinct from GMA, it is hypothesized that character strengths exhibit incremental validity beyond GMA.

The incremental validity of character strengths beyond the Big Five is of interest as well, because both character strengths and the Big Five describe individuals' personality traits. The question is whether or not character strengths—as the more recent conceptualization of personality traits—add new information to the prediction of job performance beyond the Big Five. Character strengths differ from personality traits, such as the Big Five, in several aspects. Firstly, character strengths are narrow traits, whereas the Big Five are broader. Secondly, positively valued, desirable traits were intentionally excluded from the Big Five approach, as Allport (1937) regarded character traits (i.e., valued traits) as unnecessary to describe personality. The question arises whether or not character strengths as morally valued traits add information beyond the Big Five that are by definition neutral, descriptive, non-evaluative traits (Allport, 1937). Thirdly, character strengths fulfill a number of criteria (e.g., they are valued across cultures and contribute to living a fulfilled life; Peterson and Seligman, 2004) that are not equally applicable to the Big Five traits. Nevertheless, some character strengths do meaningfully overlap with selected Big Five traits (e.g., perseverance as a character strength and conscientiousness as a Big Five trait), but the size of the correlation coefficients indicates that the concepts are unique despite some overlapping aspects (e.g., Macdonald et al., 2008; Noftle et al., 2011; McGrath et al., 2020). Fourthly, some character strengths go beyond the traditional Big Five (especially those related to transcendental strengths). Therefore, as character strengths show substantial relations with various dimensions of job performance and are largely theoretically and empirically distinct from the Big Five, it is hypothesized that character strengths exhibit incremental validity beyond the Big Five.

### The Present Study

The present study aimed at examining the following research questions: do character strengths predict a significant amount of variance in job performance beyond GMA and the Big Five? Which predictors among character strengths, GMA, and the Big Five are the most important ones?

Therefore, the main goal of the present study was the investigation of the incremental validity of character strengths as predictors of job performance beyond (a) GMA, (b) the Big Five, and (c) GMA and the Big Five combined by utilizing step-wise regression analyses. Additionally, we aimed at identifying the most important predictors of job performance out of the 24 character strengths, GMA, and the Big Five by utilizing exploratory relative weight analyses. This would also provide relevant information on the relative importance of the character strengths vs. GMA vs. the Big Five in the prediction of job performance.

A sample of employees from various occupations has been collected in order to examine the goals of the present study on a more general instead of a job-specific level. In line with well-known meta-analyses (Schmidt and Hunter, 1998; e.g., Salgado and Anderson, 2003), GMA and the Big Five were conceptualized on a broad level in the present study. In order to achieve a fine-grained overview of the interplay between character strengths as narrow traits and job performance, (1) a measure based on the positive work role performance model by Griffin et al. (2007) was utilized to assess productive work behavior and its dimensions on different levels of abstraction from broad (i.e., overall job performance) to narrow (e.g., individual task proficiency). Furthermore, (2) counterproductive behavior was operationalized using a measure of deviant behavior at work and its dimensions interpersonal deviance and organizational deviance (Bennett and Robinson, 2000).

We decided to combine supervisory ratings for the dimensions of job performance with self-ratings of character strengths and the Big Five as well as test data for GMA to control for inflated correlations due to common method variance (Doty and Glick, 1998). Utilizing only supervisory ratings, self-descriptions, or test data may lead to inflated correlation coefficients.

## Materials and Methods

### Procedure and Participants

In order to obtain a heterogeneous, ideally representative sample of German employees, supervisors from various companies and sectors (e.g., air traffic and air traffic control, counseling, engineering, finance, health care, IT, craftsmen) were recruited for participation. Supervisors were informed about the study directly and using the snowball system *via* email and social networks (e.g., Xing, LinkedIn). Once supervisors and their employees decided to participate, the supervisors registered themselves and their employees by providing everyone's email address in an online registration form created using the Internet platform Unipark (http://www.unipark.com/en/). Automatically generated individual links to an anonymized online survey (also created using the Internet platform Unipark) were then sent by email to each of the employees to obtain their self-ratings in character strengths and the Big Five and to the supervisors to obtain supervisor ratings of the employees' productive and counterproductive behavior. At the end of the online survey, employees were instructed to follow a link to the Hogrefe Test System in order to complete the test of GMA. Before filling out the online survey, employees received basic information regarding the study and subsequently expressed their (dis)interest of participation (i.e., informed consent). Participants did not receive any payment for their participation, but employees had the opportunity to receive automatically generated individual feedback on their character strengths as well as extensive material on interpreting and processing the feedback. Employees and supervisors filled out the online surveys independently of each other and did not have access to each other's answers. Both the employees and the supervisors were informed about this in advance.

The sample of *employees* consisted of *N* = 169 German-speaking participants (male: *n* = 94; female: *n* = 75) from various occupational groups. The participants' mean age was *M* = 38.36 years (*SD* = 9.01, ranging from 22 to 61 years). They were highly educated, as *n* = 71 indicated having a university degree (i.e., bachelor's or master's) and *n* = 18 a doctoral degree; *n* = 75 had completed an apprenticeship, and *n* = 5 had finished secondary school. Their average length of tenure in the occupation was *M* = 10.97 years (*SD* = 7.99, ranging from 0.33 to 39.96 years). The participants were all working at least 50% of full-time hours, with about three quarters (*n* = 131) working full-time and *n* = 35 working part-time (i.e., 50–85% of a full-time position); *n* = 3 did not respond to the question. The gender distribution, average age, and share of full-time and part-time workers in the present sample were very similar to that of the German workforce as a whole, but the education of the present sample of employees was higher than on the population level (Statistisches Bundesamt, 2018; Bundesagentur für Arbeit, 2019).

The sample of *supervisors* consisted of *N* = 27 German-speaking participants (male: *n* = 19; female: *n* = 8) with a mean age of *M* = 46.26 years (*SD* = 7.35, ranging from 33 to 56 years). Each supervisor rated 1–13 employees (*M* = 6.26, *SD* = 2.70, *Md* = 7.00). The mean rating for how well they know their employees (1 = *not at all* to 5 = *partially* and 9 = *very well*) was *M* = 7.48 (*SD* = 1.37, ranging from 5 to 9). They had known their employees for *M* = 6.31 years on average (*SD* = 2.76, ranging from 1.83 to 13.17 years). This indicates that the supervisors knew their employees very well and were therefore able to judge their behavior at the workplace.

### Measures

#### Employees' Self-Assessments

##### Character Strengths

For the self-assessment of 24 character strengths, the German version of the *Values in Action Inventory of Strengths* (Peterson et al., 2005; German version: Ruch et al., 2010; VIA-IS) was utilized in its 120-item short form (VIA-IS120; Littman-Ovadia, 2015). This short form comprises five items for each of the 24 character strengths in the VIA classification (Peterson and Seligman, 2004). Participants rated the extent to which each item describes them well on a 5-point answer scale ranging from 1 = *not like me at all* to 5 = *very much like me*. For example, the character strength of perseverance is measured by items, such as “I never quit a task before it is done.” Reliability of the VIA-IS120 scales ranged from α = 0.64 to α = 0.90, with a median of α = 0.78 (Littman-Ovadia, 2015). The relations between the short form scales and the longer 240-item form scales ranged from *r* = 0.84 (honesty) to *r* = 0.96 (hope and teamwork) (Littman-Ovadia, 2015), which indicates satisfactory construct validity. For the purpose of the present study, 24 variables were computed by calculating the mean of the respective items, which represent the participants' levels of each of the 24 character strengths.

##### General Mental Ability

For the self-assessment of GMA, the short form (Part 1) of the *Revised Culture Fair Intelligence Test Scale 2* (*CFT 20-R*; Weiß, 2006) was utilized in its computer-based version (i.e., Hogrefe Test System; www.testzentrale.de/etesting/hogrefe-testsystem-hts). The CFT 20-R assesses fluid intelligence using 56 items grouped into four types of non-verbal figural tasks (i.e., 15 series, 15 classifications, 15 matrices, 11 topologies). Answers to the tasks were given in multiple-choice format and under time-limited conditions (i.e., 4 min for series and classifications and 3 min for matrices and topologies). The short form of the CFT 20-R showed a split-half reliability of *r* = 0.90 (Weiß, 2006). All four types of non-verbal figural tasks showed high loadings on a general fluid ability factor, indicating the factorial validity of the CFT 20-R (Weiß, 2006). Additionally, the CFT 20-R showed convergent validity, as it substantially correlated with other measures of intelligence (Weiß, 2006). For the purpose of the present study, one variable was computed as the number of correct answers (i.e., raw score) to represent participants' level of GMA (GMA).

##### Big Five

For the self-assessment of the Big Five personality traits neuroticism (N), extraversion I, culture (Cu), agreeableness (A), and conscientiousness (Co), the *Minimal Redundancy Scales-*−*25* (*MRS-25*; Ostendorf, 1990; Schallberger and Venetz, 1999) was utilized. This measure is based on the lexical approach research tradition (e.g., Ostendorf, 1990). The MRS-25 comprises a total of 25 items presented as bipolar adjective ratings (i.e., five items for each of the five personality factors). Participants rated the extent to which each item describes them well on a six-point bipolar rating-scale (1 = *strongly agree with the adjective on the left pole* to 6 = *strongly agree with the adjective on the right pole*). Sample items are “hardy vs. vulnerable” (N), “talkative vs. silenI(E), “original vs. conventional” (Cu), “peaceable vs. quarrelsome” (A), and “ambitious vs. aimless” (Co). The MRS-25 was found to be a reliable instrument (e.g., median of α = 0.81 in four different samples; Schallberger and Venetz, 1999). Furthermore, its stable factor structure provides strong evidence of factorial validity (Schallberger and Venetz, 1999). Although research on its construct validity is relatively scarce yet, studies have shown meaningful correlation pattern with other personality constructs (e.g., Schallberger and Venetz, 1999; Ruch et al., 2018). For the purpose of the present study, five variables were computed by calculating the means of the respective items, which represent the participants' levels of the five Big Five personality traits (i.e., neuroticism, extraversion, culture, agreeableness, conscientiousness).

#### Supervisor Ratings of Employees' Job Performance

##### Productive Work Behavior

For supervisor ratings of employees' productive work behaviors, the *Work Role Performance Scale* (*WRPS*; Griffin et al., 2007; German version: Harzer et al., 2017) was utilized. Employees' productive work behaviors are measured at three levels (i.e., individual, team, and organization level) with respect to three different aspects (i.e., proficiency, adaptivity, and proactivity). The WRPS comprises 27 items assessing the dimensions of performance in a specific work role as stipulated in the model of positive work role behaviors by Griffin et al. (2007). The supervisors are asked to rate how often their employees had carried out the described behavior in the last 1 year on a 5-point answer scale ranging from 1 = *(almost) never* to 5 = *very often*. For example, the individual task proficiency is measured by items, such as “He/she has carried out the core parts of his/her job well,” and team member adaptivity is measured by items, such as “He/she has responded constructively to changes in the way his/her team works.” Harzer et al. (2017) reported reliabilities ranging from α = 0.73 (proficiency and individual level performance) to α = 0.92 (proactivity) and α = 0.90 for overall performance. For the purpose of the present study and in accordance with Figures 1, 13 variables have been computed by calculating the means of the respective items, which represent the participants' levels in (1) individual task proficiency, (2) individual task adaptivity, (3) individual task proactivity, (4) team member proficiency, (5) team member adaptivity, (6) team member proactivity, (7) organization member proficiency, (8) organization member adaptivity, (9) organization member proactivity, (10) individual-level performance (i.e., composite score of individual task proficiency, adaptivity, and proactivity), (11) team-level performance (i.e., composite score of team member proficiency, adaptivity, and proactivity), (12) organization-level performance (i.e., composite score of organization member proficiency, adaptivity, and proactivity), and (13) overall job performance (i.e., composite score of all dimensions [1] to [9]).

##### Counterproductive Work Behavior

For supervisor ratings of employees' counterproductive work behavior, the *Workplace Deviance Scale* (*WDS*; Bennett and Robinson, 2000; German version: Harzer et al., 2017) was utilized. The WDS comprises 19 items assessing employees' deviant and counterproductive behaviors at the workplace. It includes the subscales of interpersonal deviance (7 items; deviant behaviors directly harmful to other individuals within the organization) and organizational deviance (12 items; deviant behaviors directly harmful to the organization). The supervisors were asked to indicate the frequency with which their employees engaged in the described behaviors over the past year on a 7-point answer scale ranging from 1 = *never* to 7 = *daily*. An example item for interpersonal deviance is “He/she made fun of someone at work” and for organizational deviance “He/she has taken property from work without permission.” Harzer et al. (2017) reported internal consistencies of α = 0.71 and α = 0.74 for interpersonal deviance and organizational deviance, respectively. For the purpose of the present study, three variables were computed by calculating the means of the respective items, which represent the employees' levels of (1) interpersonal deviance, (2) organizational deviance, and (3) overall deviant behavior at work [i.e., composite score of (1) and (2)].

#### Control Variables

Sex and age were included as control variables for two reasons. Firstly, a meta-analysis indicated systematic relations between character strengths and these demographic variables (Heintz et al., 2019). Secondly, age (as a proxy for work experience) has been shown to have an impact on job performance (e.g., Quińones et al., 1995).

### Data Screening

In order to ensure their trustworthiness and accuracy, the data were screened thoroughly. The raw data encompass 175 employees with complete data on the self-rating measures (i.e., VIA-IS120, MRS-25) and supervisor-rated measures (i.e., WRPS, WDS). A total of 6 cases were excluded from the data analyses: *n* = 2 because of answer styles and contradictory answers by the employees, *n* = 1 because the employee's sex differed between the self- and supervisor ratings, and *n* = 3 because the supervisors indicated that they did not know the evaluated employee well enough. Consequently, the final data set included *N* = 169 cases.

Furthermore, there was substantial dropout on the CFT 20-R data, because employees needed to change to a different online platform after filling out the self-assessment measures in order to complete the CFT 20-R (i.e., from the Unipark to the Hogrefe Test System). As some employees did not do so, CFT 20-R scores were available for 106 of the 169 cases. However, employees who filled out the CFT 20-R did not differ significantly from those who did not complete the CFT 20-R with respect to gender ratio [χ^2^(1) = 0.00, *p* = 0.989], age [*t*_(167)_ = −1.02, *p* = 0.310], education [χ^2^(5) = 9.15, *p* = 0.103], tenure [*t*_(167)_ = 0.18, *p* = 0.862], or any of the measures from the self- and supervisor ratings [$V_{Pillai^{\prime }sTrace}$ = 0.28, *F*_*MANOVA*__(41,127)_ = 1.22, *p* = 0.199]. Additionally, Little's MCAR test indicated that the data were missing completely at random [χ^2^(48) = 56.40, *p* = 0.190]. Therefore, using the R package “mice,” incomplete data were imputed *via* chained equations (van Buuren and Groothuis-Oudshoorn, 2011). A total of 40 data sets were imputed with 20 iterations each in order to obtain satisfactory imputations (Graham et al., 2007; Graham, 2009; van Buuren and Groothuis-Oudshoorn, 2011). Inspection of the imputed data showed that they were trustworthy (van Buuren and Groothuis-Oudshoorn, 2011): (a) imputed values were within the range of possible scores on the CFT 20-R, (b) there was high convergence among the imputed data sets, and (c) density plots of the observed and imputed CFT 20-R raw scores were highly similar. As it was not possible to work with 40 data sets for all the subsequent data analyses, these were merged into one data set utilizing the R package “sjmisc” (Lüdecke, 2018). Densities of the mean values of the 40 imputed data sets and the final merged CFT 20-R raw scores were highly similar, indicating a highly satisfactory merging process.

## Results

### Preliminary Analyses

In order to examine the utilized measures (i.e., VIA-IS120, CFT 20-R, MRS-25, WRPS, WDS), minima, maxima, means, standard deviations, and reliability coefficients (Cronbach's alpha) were computed for all scales. Furthermore, correlations between the variables and employees' sex and age were calculated (see Table 2 for employees' self-assessments and Table 3 for supervisors' ratings of their employees).

**Table 2:** Employees' self-assessment of character strengths, GMA, and the Big Five: minima, maxima, means, standard deviations, Cronbach's alpha coefficients of VIA-IS120 scales, CFT 20-R, and MRS-25 scales, and correlations between VIA-IS120 scales, CFT 20-R, and MRS-25 scales and participants' sex and age.

						**Correlation with participants'**	
**Variable**	**Min**	**Max**	***M***	***SD***	**α**	**Sex**	**Age**
**VIA-IS120**							
Creativity	1.00	5.00	2.70	0.98	0.91	0.12	0.07
Curiosity	1.20	5.00	2.98	0.94	0.91	0.20^**^	0.10
Judgment	1.60	5.00	3.41	0.80	0.89	−0.14	0.33^***^
Love of learning	1.00	5.00	2.73	1.00	0.91	0.07	0.34^***^
Perspective	1.40	4.80	2.97	0.84	0.80	−0.17^*^	0.46^***^
Bravery	1.60	5.00	3.31	0.85	0.82	−0.15^*^	0.31^***^
Perseverance	1.60	5.00	3.56	0.87	0.90	−0.09	0.31^***^
Honesty	1.80	5.00	3.65	0.71	0.77	0.24^**^	0.27^***^
Zest	1.80	5.00	3.53	0.76	0.77	−0.01	0.19^*^
Love	1.20	5.00	3.21	0.89	0.90	0.30^***^	0.13
Kindness	1.40	5.00	3.46	0.80	0.86	0.40^***^	0.11
Social intelligence	1.40	5.00	3.43	0.79	0.88	0.36^***^	0.10
Teamwork	1.40	5.00	3.56	0.86	0.88	0.30^***^	0.14
Fairness	1.40	5.00	3.76	0.90	0.91	0.22^**^	0.23^**^
Leadership	1.00	5.00	2.81	1.22	0.92	−0.04	0.44^***^
Forgiveness	1.60	5.00	3.67	0.72	0.77	0.15	0.27^***^
Modesty	1.00	5.00	3.38	0.80	0.81	0.00	0.38^***^
Prudence	1.60	5.00	3.31	0.73	0.77	0.16^*^	0.31^***^
Self-regulation	1.40	5.00	3.28	0.86	0.81	0.10	0.20^**^
Appreciation	1.00	5.00	2.61	1.05	0.94	0.65^***^	−0.11
Gratitude	1.20	4.80	3.12	0.80	0.86	0.26^**^	0.16^*^
Hope	1.60	4.80	3.43	0.64	0.65	0.13	0.12
Humor	1.00	5.00	2.99	0.88	0.87	0.04	0.10
Spirituality	1.00	5.00	2.31	1.09	0.96	0.07	0.25^**^
**CFT 20-R**							
GMA	31.00	54.00	43.54	6.17	0.89	−0.10	0.08
**MRS-25**							
Neuroticism	1.40	5.80	2.94	0.87	0.75	0.40^***^	−0.21^**^
Extraversion	1.80	6.00	4.31	1.08	0.91	0.13	−0.07
Culture	1.80	5.80	3.59	0.87	0.77	0.23^**^	−0.19^*^
Agreeableness	2.20	6.00	4.38	0.75	0.78	0.31^***^	0.08
Conscientiousness	2.80	6.00	4.68	0.70	0.84	0.05	0.21^**^

*N = 169. Sex: 1 = male, 2 = female. Edu = highest educational degree. Ten = tenure (years of work experience in the current profession). Pearson correlations between variables and sex, age, and tenure. Spearman's rho correlations between variables and highest educational degree. VIA-IS120 = Values in Action Inventory of Strengths (Littman-Ovadia, 2015). CFT 20-R = Revised Culture Fair Intelligence Test Scale 2 (Weiß, 2006). GMA = General mental ability. MRS-25 = Minimal Redundancy Scales (Ostendorf, 1990)*.

* *p < 0.05*;

** *p < 0.01*;

*** *p < 0.001*.

**Table 3:** Supervisor ratings of employees' productive and counterproductive work behavior: minima, maxima, means, standard deviations, Cronbach's alpha coefficients of WRPS and WDS scales, and correlations between WRPS and WDS scales and employees' sex and age.

						**Correlation with employees'**	
**Variable**	**Min**	**Max**	***M***	***SD***	**α**	**Sex**	**Age**
**WRPS**							
Overall job performance	1.52	4.78	3.11	0.74	0.94	−0.05	0.29^***^
Individual-level performance	1.56	5.00	3.38	0.79	0.94	−0.07	0.28^***^
Individual task proficiency	2.00	5.00	4.16	0.88	0.95	−0.09	0.28^***^
Individual task adaptivity	1.00	5.00	3.10	0.88	0.90	−0.03	0.19^*^
Individual task proactivity	1.00	5.00	2.88	0.91	0.91	−0.06	0.28^***^
Team-level performance	1.22	4.78	3.24	0.77	0.92	0.05	0.22^**^
Team member proficiency	1.33	5.00	4.05	0.87	0.92	0.14	0.10
Team member adaptivity	1.33	5.00	3.11	0.81	0.78	0.03	0.19^*^
Team member proactivity	1.00	4.67	2.55	0.95	0.91	−0.04	0.28^***^
Organization-level performance	1.22	5.00	2.71	0.81	0.93	−0.10	0.31^***^
Organization member proficiency	1.33	5.00	3.74	0.86	0.86	0.01	0.29^***^
Organization member adaptivity	1.00	5.00	2.30	0.92	0.90	−0.12	0.22^**^
Organization member proactivity	1.00	5.00	2.07	0.96	0.91	−0.15^*^	0.31^***^
**WDS**							
Overall deviant behavior at work	1.00	2.70	1.20	0.30	0.80	0.00	−0.19^*^
Interpersonal deviance	1.00	3.14	1.15	0.38	0.85	−0.17^*^	−0.02
Organizational deviance	1.00	3.17	1.25	0.38	0.75	0.12	−0.28^***^

*N = 169. Sex: 1 = male, 2 = female. Edu = highest educational degree. Ten = tenure (years of work experience in the current profession). Pearson correlations between variables and sex, age, and tenure. Spearman's rho correlations between variables and highest educational degree. WRPS = Work Role Performance Scale (Griffin et al., 2007). WDS = Workplace Deviance Scale (Bennett and Robinson, 2000)*.

* *p < 0.05*;

** *p < 0.01*;

*** *p < 0.001*.

Tables 2, 3 show that all measures demonstrated satisfactory variability with the exception of counterproductive work behavior (WDS). The minima and maxima indicated that the sample consisted of participants having low to high scores on the variables. The scale reliability coefficients were satisfactory for research purposes. As there were small- to medium-sized systematic correlations between the utilized measures and employees' sex and age, these demographic variables were included as control variables in the subsequent data analyses in order to prevent any bias in the results due to these variables. Skewness and kurtosis of all the measures indicated normal distribution for all variables except the counterproductive work behavior (WDS). The variables representing counterproductive work behavior were substantially L-shaped; therefore, they were *inversely* transformed (Tabachnick and Fidell, 2001) for further use in subsequent analyses.

To obtain an overview of the relations of character strengths, GMA, and the Big Five with productive and counterproductive work behavior, zero-order and partial correlations were computed between (a) the VIA-IS120 scales, CFT 20-R, and MRS-25 scales and (b) the WRPS scales and WDS scales. Due to the large number of correlation coefficients, a Bonferroni correction was employed to control for Type I error, conservatively adjusting the alpha level to 0.0016 (0.05/30, because there were 24 VIA-IS120 scales, 1 CFT 20-R score, and 5 MRS-25 scales). Partial correlations (control variables: employees' sex and age) are presented in Table 4 (please see Supplementary Table 1 for zero-order correlations among the study variables).

**Table 4:** Partial correlations (controlled for employees' sex and age) between employees' self-assessed character strengths, GMA, and the Big Five (VIA-IS120 scales, CFT 20-R, MRS-25 scales) and supervisor ratings of employees' productive and counterproductive work behavior (WRPS and WDS scales).

	**WRPS**													**WDS**		
		**Individual task**				**Team member**				**Organization member**						
**Variable**	**Overall**	**Total**	**Prof**	**Adapt**	**Proact**	**Total**	**Prof**	**Adapt**	**Proact**	**Total**	**Prof**	**Adapt**	**Proact**	**Overall**	**Int**	**Org**
**VIA-IS120**																
Creativity	0.37	0.41	0.29	0.44	0.34	0.33	0.19	0.39	0.30	0.30	0.29	0.32	0.19	−0.24	−0.01	−0.35
Curiosity	0.38	0.39	0.25	0.41	0.36	0.32	0.16	0.33	0.35	0.34	0.29	0.37	0.25	−0.14	0.02	−0.20
Judgment	0.52	0.56	0.55	0.52	0.42	0.44	0.28	0.46	0.41	0.45	0.44	0.43	0.31	−0.32	−0.07	−0.42
Love of learning	0.45	0.41	0.29	0.38	0.40	0.44	0.31	0.41	0.44	0.40	0.38	0.34	0.34	−0.30	−0.21	−0.26
Perspective	0.48	0.44	0.44	0.42	0.31	0.46	0.38	0.44	0.38	0.45	0.46	0.39	0.34	−0.44	−0.21	−0.46
Bravery	0.47	0.53	0.49	0.54	0.36	0.36	0.20	0.42	0.32	0.43	0.44	0.42	0.30	−0.30	−0.04	−0.43
Perseverance	0.63	0.68	0.73	0.61	0.46	0.53	0.43	0.54	0.43	0.55	0.59	0.51	0.37	−0.48	−0.16	−0.58
Honesty	0.61	0.53	0.56	0.48	0.37	0.59	0.55	0.59	0.42	0.57	0.57	0.52	0.42	−0.49	−0.25	−0.52
Zest	0.45	0.49	0.47	0.47	0.36	0.40	0.34	0.38	0.32	0.38	0.47	0.36	0.20	−0.38	−0.12	−0.45
Love	0.32	0.27	0.27	0.24	0.19	0.39	0.39	0.36	0.27	0.23	0.29	0.18	0.16	−0.38	−0.24	−0.34
Kindness	0.52	0.42	0.44	0.40	0.27	0.57	0.57	0.55	0.39	0.45	0.48	0.41	0.32	−0.49	−0.27	−0.47
Social intelligence	0.50	0.40	0.46	0.32	0.28	0.60	0.64	0.54	0.41	0.41	0.49	0.33	0.29	−0.53	−0.36	−0.46
Teamwork	0.64	0.52	0.56	0.44	0.37	0.74	0.80	0.66	0.49	0.53	0.60	0.45	0.38	−0.63	−0.38	−0.58
Fairness	0.60	0.52	0.52	0.47	0.38	0.63	0.60	0.58	0.47	0.53	0.57	0.45	0.39	−0.57	−0.35	−0.54
Leadership	0.66	0.52	0.44	0.49	0.43	0.61	0.46	0.61	0.53	0.73	0.63	0.65	0.65	−0.44	−0.20	−0.45
Forgiveness	0.49	0.41	0.39	0.39	0.30	0.54	0.54	0.48	0.41	0.41	0.50	0.34	0.27	−0.54	−0.38	−0.45
Modesty	0.32	0.31	0.29	0.31	0.21	0.34	0.35	0.29	0.24	0.25	0.33	0.23	0.11	−0.28	−0.12	−0.32
Prudence	0.33	0.37	0.34	0.37	0.26	0.29	0.19	0.32	0.25	0.28	0.33	0.28	0.13	−0.17	−0.03	−0.22
Self-regulation	0.41	0.46	0.48	0.41	0.32	0.36	0.33	0.33	0.29	0.32	0.40	0.28	0.18	−0.40	−0.19	−0.42
Appreciation	0.11	0.06	0.07	0.07	0.03	0.16	0.18	0.16	0.08	0.09	0.15	0.06	0.03	−0.19	−0.12	−0.13
Gratitude	0.32	0.32	0.36	0.27	0.23	0.37	0.38	0.36	0.24	0.21	0.31	0.19	0.07	−0.37	−0.23	−0.32
Hope	0.44	0.42	0.42	0.35	0.33	0.42	0.34	0.40	0.37	0.39	0.48	0.30	0.26	−0.40	−0.21	−0.40
Humor	0.48	0.42	0.31	0.47	0.31	0.43	0.30	0.45	0.38	0.50	0.45	0.46	0.42	−0.31	−0.07	−0.37
Spirituality	0.09	0.10	0.10	0.05	0.12	0.12	0.12	0.08	0.10	0.04	0.06	0.04	0.02	−0.14	−0.09	−0.09
**CFT 20-R**																
GMA	0.39	0.44	0.36	0.41	0.39	0.37	0.34	0.32	0.31	0.27	0.22	0.24	0.26	−0.27	−0.05	−0.33
**MRS-25**																
Neuroticism	−0.27	−0.28	−0.28	−0.22	−0.24	−0.25	−0.21	−0.18	−0.26	−0.21	−0.22	−0.13	−0.22	0.32	0.18	0.31
Extraversion	0.30	0.23	0.34	0.11	0.14	0.35	0.45	0.26	0.21	0.27	0.33	0.19	0.22	−0.45	−0.26	−0.43
Culture	0.32	0.33	0.30	0.33	0.23	0.30	0.28	0.30	0.22	0.26	0.27	0.23	0.19	−0.36	−0.19	−0.38
Agreeableness	0.34	0.29	0.35	0.23	0.18	0.38	0.43	0.32	0.24	0.30	0.33	0.26	0.21	−0.38	−0.30	−0.30
Conscientiousness	0.40	0.36	0.41	0.32	0.22	0.37	0.34	0.36	0.27	0.38	0.37	0.34	0.30	−0.36	−0.15	−0.40

*N = 169. WRPS = Work Role Performance Scale (Griffin et al., 2007): Overall = Overall job performance, Total = Composite score of 3 respective scales, Prof = Proficiency, Adapt = Adaptivity, Proact = Proactivity. WDS = Workplace Deviance Scale (Bennett and Robinson, 2000): Overall = Overall deviant behavior at work, Int = Interpersonal deviance, Org = Organizational deviance. VIA-IS120 = Values in Action Inventory of Strengths (Littman-Ovadia, 2015). R^2^ = multiple correlation coefficient including all character strengths that were significantly related at p < 0.0016; CFT 20-R = Revised Culture Fair Intelligence Test Scale 2 (Weiß, 2006); GMA = general mental ability; MRS-25 = Minimal Redundancy Scales (Ostendorf, 1990). Significance cut-off: correlation coefficients ≥|0.25| were significant at p < 0.0016*.

Table 4 shows that there were numerous significant positive correlations between character strengths and the dimensions of productive work behavior as well as negative correlations between character strengths and counterproductive work behavior. Due to high variability in the data and the high reliability of the scales, correlation coefficients representing the relations between character strengths and the various dimensions of job performance were higher than in previous research, but the correlation patterns were similar (e.g., Cosentino and Castro Solano, 2012; Harzer and Ruch, 2014; Littman-Ovadia and Lavy, 2016; Harzer et al., 2017). More specifically, perseverance, teamwork, and leadership most often exhibited the numerically highest correlation coefficients within each of the columns of Table 4. Perseverance showed the numerically strongest correlations with (the dimensions of) individual-level performance, teamwork with (the dimensions of) team-level performance as well as (the dimensions of) counterproductive work behavior, and leadership with (the dimensions of) organization-level performance. However, other character strengths were numerically strong correlates of various dimensions of productive and counterproductive work behavior as well (e.g., honesty for individual task proficiency, social intelligence for team member proficiency, fairness and forgiveness for counterproductive behavior). Furthermore, the character strengths were numerically less strongly related to interpersonal deviance than the other dimensions of productive and counterproductive work behavior.

The effect size of the correlation between GMA and overall job performance was similar to those reported in meta-analyses (Schmidt and Hunter, 1998; Salgado et al., 2003; e.g., Hülsheger et al., 2007). Furthermore, the correlations between the Big Five and job performance were stronger than those reported in meta-analyses (e.g., Barrick and Mount, 1991; Salgado, 1997; Hurtz and Donovan, 2000; Dudley et al., 2006). Nevertheless, in line with the results of these meta-analyses, conscientiousness most often exhibited the numerically strongest relations to the dimensions of productive work behavior among the Big Five.

### Regression Analyses

In order to examine the incremental validity of character strengths as predictors of job performance beyond GMA and the Big Five, several hierarchical linear regression analyses were computed. The R package “personality factors” was utilized to estimate Olkin–Pratt adjusted *R*^2^ and Δ*R*^2^, which is recommended for regression models with largely different numbers of predictors and collinearity among predictors (Anglim and Grant, 2014). *Firstly*, we were interested in the incremental validity of character strengths as predictors of job performance beyond GMA. Therefore, a hierarchical linear regression analysis was computed for each of the dimensions of productive and counterproductive work behavior (controlling for sex and age^1^) as the dependent variable. In the first step, CFT 20-R raw scores (controlling for sex and age) were entered as independent variables (method: Enter), whereas in the second step, those variables among the VIA-IS120 scales (controlling for sex and age) that were significantly related to the dependent variable of interest (as presented in Table 4) were entered as independent variables (method: Enter). Changes in the explained variance (Olkin–Pratt adjusted Δ*R*^2^) of the dependent variables from Step 1 to Step 2 were of particular interest. If there was a significant increase in the explained variance, character strengths exhibited incremental validity beyond GMA (and the control variables sex and age).

*Secondly*, we were interested in the incremental validity of character strengths as predictors of job performance beyond the Big Five. The logic and analysis procedure were congruent with the regression analyses examining the incremental validity of character strengths beyond GMA. However, in Step 1, the MRS-25 scales (controlling for sex and age) were entered as independent variables (method: Enter) instead of CFT 20-R raw scores (controlling for sex and age).

*Thirdly*, we were interested in the incremental validity of character strengths as predictors of job performance beyond GMA and the Big Five combined. Therefore, in Step 1, the CFT 20-R raw scores and MRS-25 scales (all controlling for sex and age) were entered as independent variables (method: Enter). Tables 5–**7** present the results of the hierarchical linear regression analyses examining the incremental validity of character strengths as predictors of job performance beyond GMA, the Big Five, as well as GMA plus the Big Five, respectively.

**Table 5:** Hierarchical linear regression analyses: explained variance (Olkin–Pratt adjusted) in dependent variables by GMA (Step 1; method: Enter; CFT 20-R) and character strengths (Step 2; method: Enter; VIA-IS120 scales with partial correlation coefficients ≥0.25 in accordance with Table 4).

	**Step 1: GMA**	**Step 2: Character strengths**	
**Dependent variable**	***R*^2^**	**Δ*R*^2^**	**Total *R*^2^**
**WRPS**			
Overall job performance	0.146	0.508	0.654
Individual-level performance	0.191	0.398	0.588
Individual task proficiency	0.126	0.489	0.615
Individual task adaptivity	0.161	0.362	0.523
Individual task proactivity	0.152	0.205	0.356
Team-level performance	0.134	0.507	0.641
Team member proficiency	0.111	0.532	0.643
Team member adaptivity	0.099	0.477	0.576
Team member proactivity	0.092	0.286	0.379
Organization-level performance	0.068	0.548	0.616
Organization member proficiency	0.042	0.510	0.552
Organization member adaptivity	0.054	0.454	0.508
Organization member proactivity	0.061	0.397	0.458
**WDS**			
Overall deviant behavior at work	0.066	0.390	0.456
Interpersonal deviance	0.000	0.155^ns^	0.155
Organizational deviance	0.107	0.327	0.434

*N = 169. All data were corrected for effects of sex and age before being entered into the regression analyses. GMA = general mental ability; CFT 20-R = Revised Culture Fair Intelligence Test Scale 2 (Weiß, 2006)*.

Δ*R^2^ = incrementally explained variance; p = significance level; WRPS = Work Role Performance Scale (Griffin et al., 2007); WDS = Workplace Deviance Scale (Bennett and Robinson, 2000). Only character strengths that showed a significant correlation (p < 0.0016) with the dimension of productive or counterproductive work behavior of interest were considered here*.

ns *= ΔR^2^ was not statistically significant*.

Overall, the results of the regression analyses indicated that character strengths exhibited incremental validity as predictors of all dimensions of productive and counterproductive work behavior beyond GMA and/or the Big Five (except interpersonal deviance). The results of the regression analyses with respect to the interpersonal deviance outcome need to be treated with caution, as the residuals did not exhibit a normal distribution.

More specifically, Table 5 shows that explained variance in the dependent variables (except interpersonal deviance) significantly increased by between 20.5 (individual task proactivity) and 54.8% (organization-level performance) by adding character strengths as independent variables in addition to GMA. GMA explained up to 19.1% of the variance in the dependent variables. Table 6 shows that explained variance in the dependent variables (except interpersonal deviance) significantly increased by between 16.2 (overall deviant behavior at work) and 43.1% (organization-level performance) by adding character strengths as independent variables in addition to the Big Five. The Big Five explained up to 32.4% of the variance in the dependent variables. Table 7 shows that explained variance in the dependent variables (except interpersonal deviance) significantly increased by between 10.7 (organizational deviance) and 38.4% (organization-level performance) by adding character strengths as independent variables in addition to GMA and the Big Five. GMA and the Big Five combined explained up to 37.5% of the variance in the dependent variables.

**Table 6:** Hierarchical linear regression analyses: explained variance (Olkin–Pratt adjusted) in dependent variables by the Big Five (Step 1; method: Enter; MRS-25 scales) and character strengths (Step 2; method: Enter; VIA-IS120 scales with partial correlation coefficients ≥0.25 in accordance with Table 4).

	**Step 1: Big Five**	**Step 2: Character strengths**	
**Dependent variable**	***R*^2^**	**Δ*R*^2^**	**Total *R*^2^**
**WRPS**			
Overall job performance	0.237	0.410	0.647
Individual-level performance	0.204	0.368	0.572
Individual task proficiency	0.255	0.353	0.608
Individual task adaptivity	0.179	0.341	0.521
Individual task proactivity	0.086	0.235	0.321
Team-level performance	0.241	0.393	0.634
Team member proficiency	0.295	0.337	0.632
Team member adaptivity	0.189	0.388	0.577
Team member proactivity	0.112	0.265	0.377
Organization-level performance	0.182	0.431	0.613
Organization member proficiency	0.205	0.325	0.530
Organization member adaptivity	0.133	0.376	0.509
Organization member proactivity	0.102	0.347	0.449
**WDS**			
Overall deviant behavior at work	0.324	0.162	0.486
Interpersonal deviance	0.110	0.040^ns^	0.149
Organizational deviance	0.310	0.171	0.480

*N = 169. All data were corrected for effects of sex and age before being entered into the regression analyses. MRS-25 = Minimal Redundancy Scales (Ostendorf, 1990). ΔR^2^ = incrementally explained variance; p = significance level; WRPS = Work Role Performance Scale (Griffin et al., 2007); WDS, Workplace Deviance Scale (Bennett and Robinson, 2000). Only character strengths that showed a significant correlation (p < 0.0016) with the dimension of productive or counterproductive work behavior of interest were considered here*.

ns *= ΔR^2^ was not statistically significant*.

**Table 7:** Hierarchical linear regression analyses: explained variance in dependent variables (Olkin–Pratt adjusted) by GMA and the Big Five (Step 1; method: Enter; CFT 20-R and MRS-25 scales) and character strengths (Step 2; method: Enter; VIA-IS120 scales with partial correlation coefficients ≥0.25 in accordance with Table 4).

	**Step 1: GMA and Big Five**	**Step 2: Character strengths**	
**Dependent variable**	***R*^2^**	**Δ*R*^2^**	**Total *R*^2^**
**WRPS**			
Overall job performance	0.341	0.310	0.651
Individual-level performance	0.345	0.237	0.582
Individual task proficiency	0.342	0.264	0.606
Individual task adaptivity	0.298	0.234	0.532
Individual task proactivity	0.205	0.144	0.349
Team-level performance	0.336	0.306	0.642
Team member proficiency	0.375	0.275	0.650
Team member adaptivity	0.258	0.322	0.580
Team member proactivity	0.179	0.200	0.379
Organization-level performance	0.226	0.384	0.610
Organization member proficiency	0.227	0.314	0.542
Organization member adaptivity	0.170	0.335	0.506
Organization member proactivity	0.144	0.316	0.460
**WDS**			
Overall deviant behavior at work	0.356	0.127	0.483
Interpersonal deviance	0.104	0.044^ns^	0.148
Organizational deviance	0.370	0.107	0.477

*N = 169. All data were corrected for effects of sex and age before being entered into the regression analyses. GMA = general mental ability; CFT 20-R = Revised Culture Fair Intelligence Test Scale 2 (Weiß, 2006); MRS-25 = Minimal Redundancy Scales (Ostendorf, 1990); ΔR^2^ = incrementally explained variance; p = significance level; WRPS = Work Role Performance Scale (Griffin et al., 2007); WDS = Workplace Deviance Scale (Bennett and Robinson, 2000). Only character strengths that showed a significant correlation (p < 0.0016) with the dimension of productive or counterproductive work behavior of interest were considered here*.

ns *= ΔR^2^ was not statistically significant*.

### Relative Weight Analyses

Because relative weight analyses adequately take into account the multicollinearity of predictors (Johnson, 2000; Tonidandel and LeBreton, 2015), they were conducted to explore the relative importance of the job performance predictors of interest in the present study (i.e., 24 character strengths, GMA, 5 Big Five). The relative weight analyses were computed using RWA-web (Tonidandel and LeBreton, 2015) to obtain an overview of significant predictors of the various dimensions of job performance. The predictors were sex- and age-corrected VIA-IS120 scales, CFT 20-R raw scores, and MRS-25 scales. As recommended by Tonidandel et al. (2009) as well as Tonidandel and LeBreton (2015), confidence intervals for the relative weights of the predictors and significance tests were based on 10,000 bootstrapped samples, and bias-corrected and accelerated 95% confidence intervals were used. Results from these analyses are presented in Table 8 for overall job performance, individual-level performance, team-level performance, and organization-level performance (WRPS) as well as overall deviant behavior at work (WDS). Results for the more fine-grained subdimensions of productive and counterproductive work behavior (i.e., individual task, team member, and organization member proficiency, adaptivity, and proactivity, respectively; interpersonal and organizational deviance) are presented in Supplementary Table 2.

**Table 8:** Relative weights (RW) and percentages of explained criterion variance (%) for all character strengths, GMA, and the Big Five (VIA-IS120 scales, CFT 20-R, and MRS-25 scales) for overall job performance, individual-level performance, team-level performance, and organization-level performance (WRPS scales) as well as overall deviant behavior at work (WDS).

	**WRPS**								**WDS**	
	**Overall job performance**		**Individual-level performance**		**Team-level performance**		**Organ.-level performance**		**Overall**	
**Predictor**	**RW**	**%**	**RW**	**%**	**RW**	**%**	**RW**	**%**	**RW**	**%**
**VIA-IS120**										
Creativity	0.020^*^	2.8	0.031	4.6	0.016^*^	2.3	0.009	1.4	0.006	1.0
Curiosity	0.014	1.9	0.016	2.5	0.008	1.1	0.014	2.1	0.007	1.2
Judgment	0.036^*^	5.0	0.050^*^	7.6	0.023^*^	3.3	0.026^*^	3.8	0.013	2.3
Love of learning	0.035^*^	4.9	0.031	4.6	0.037^*^	5.2	0.026^*^	3.9	0.011	1.8
Perspective	0.020^*^	2.7	0.015	2.2	0.017^*^	2.4	0.024^*^	3.5	0.028	4.7
Bravery	0.019^*^	2.6	0.029	4.3	0.008	1.2	0.019^*^	2.7	0.008	1.3
Perseverance	0.054^*^	7.4	0.080^*^	12.0	0.028^*^	4.0	0.041^*^	6.1	0.030	5.0
Honesty	0.033^*^	4.6	0.024	3.6	0.033^*^	4.6	0.038^*^	5.5	0.021	3.5
Zest	0.019^*^	2.7	0.029	4.4	0.011	1.6	0.014	2.1	0.017	2.8
Love	0.008	1.0	0.005	0.8	0.014	1.9	0.005	0.7	0.016	2.7
Kindness	0.026^*^	3.6	0.016	2.3	0.036^*^	5.1	0.022^*^	3.2	0.025	4.2
Social intelligence	0.022^*^	3.0	0.011	1.7	0.044^*^	6.2	0.015	2.2	0.027	4.6
Teamwork	0.062^*^	8.7	0.034^*^	5.2	0.112^*^	15.8	0.037^*^	5.5	0.065^*^	10.8
Fairness	0.028^*^	3.8	0.019	2.8	0.034^*^	4.8	0.024^*^	3.5	0.035^*^	5.9
Leadership	0.108^*^	15.0	0.049^*^	7.4	0.069^*^	9.7	0.191^*^	28.1	0.020	3.4
Forgiveness	0.019^*^	2.7	0.011	1.6	0.029^*^	4.1	0.015	2.2	0.040^*^	6.7
Modesty	0.007	0.9	0.006	0.9	0.009	1.3	0.005	0.7	0.010	1.6
Prudence	0.010	1.4	0.015	2.2	0.007	1.0	0.008	1.1	0.012	2.0
Self-regulation	0.013	1.8	0.021	3.2	0.010	1.3	0.007	1.0	0.029	4.8
Appreciation	0.007	1.0	0.010	1.5	0.005	0.7	0.006	0.9	0.003	0.5
Gratitude	0.009	1.3	0.013	1.9	0.017^*^	2.4	0.003	0.5	0.019	3.2
Hope	0.013^*^	1.8	0.013	1.9	0.011	1.6	0.013	1.9	0.011	1.8
Humor	0.030^*^	4.2	0.018	2.7	0.021^*^	3.0	0.045^*^	6.6	0.008	1.3
Spirituality	0.001	0.2	0.002	0.2	0.002	0.2	0.001	0.2	0.002	0.3
**CFT 20-R**										
GMA	0.035^*^	4.9	0.054^*^	8.1	0.037^*^	5.2	0.012	1.8	0.010	1.6
**MRS-25**										
Neuroticism	0.011	1.5	0.014	2.1	0.011	1.5	0.005	0.7	0.031	5.2
Extraversion	0.011	1.5	0.006	0.9	0.015	2.2	0.011	1.6	0.036^*^	6.0
Culture	0.012	1.6	0.017	2.5	0.009	1.2	0.007	1.1	0.026	4.4
Agreeableness	0.015^*^	2.0	0.010	1.5	0.017^*^	2.5	0.013	1.9	0.016	2.7
Conscientiousness	0.022^*^	3.1	0.017	2.6	0.018^*^	2.6	0.023	3.4	0.016	2.6
***R***^**2**^	0.719	100	0.664	100	0.708	100	0.681	100	0.595	100

*N = 169. All data were corrected for effects of sex and age before being entered into the regression analyses. RW = raw relative weight (within rounding error, raw weights sum up to R^2^); % = relative weight rescaled to as a percentage of predicted variance in the criterion attributed to each predictor (within rounding error, rescaled weights sum to 100); WRPS = Work Role Performance Scale (Griffin et al., 2007); WDS = Workplace Deviance Scale (Bennett and Robinson, 2000): Overall = overall deviant behavior at work; VIA-IS120 = Values in Action Inventory of Strengths (Littman-Ovadia, 2015); CFT 20-R = Revised Culture Fair Intelligence Test Scale 2 (Weiß, 2006); GMA = general mental ability; MRS-25 = Minimal Redundancy Scales (Ostendorf, 1990)*.

* *95% confidence interval did not include zero (p < 0.05)*.

Table 8 shows that the combination of the predictors explained between 59.5 and 71.9% of the variance in overall job performance, individual-level performance, team-level performance, and organization-level performance as well as overall deviant behavior at work. Explained variance in the more fine-grained subdimensions of productive and counterproductive work behavior ranged between 34.1 (interpersonal deviance) and 71.9% (team member proficiency) (see Supplementary Table 2). However, none of the predictors exhibited a significant relative weight for interpersonal deviance, which might have been due to the lack of normality of the residuals; therefore, the results with respect to interpersonal deviance should be treated with caution.

Up to 16 of the 24 character strengths were significant predictors of the various dimensions of job performance (except for interpersonal deviance). GMA was a significant predictor for overall job performance, individual-level performance and its subdimensions (i.e., individual task proficiency, adaptivity, proactivity), team-level performance and its subdimensions (i.e., team member proficiency, adaptivity, proactivity), and organizational deviance, but not for organization-level performance and its subdimensions, overall deviant behavior at work or interpersonal deviance. Among the Big Five, conscientiousness followed by agreeableness and extraversion were particularly relevant predictors for the various dimensions of job performance.

For each of the dimensions of job performance, at least one character strength explained a numerically larger amount of variance than GMA and the Big Five, with the exception of individual task proactivity, where GMA exhibited the numerically highest amount of explained variance (see Table 8 and Supplementary Table 2). To conduct an exploratory investigation of the most relevant predictors among the character strengths, we took a closer look at which character strengths had a significant relative weight and a percentage of predicted variance ≥5%. Some of the character strengths seemed to be relevant more often than others. For example, teamwork explained up to 21.8% of the variance in the dimensions of job performance (except individual task adaptivity and proactivity, organization member adaptivity and proactivity, interpersonal deviance). Furthermore, leadership explained up to 34.4% of the variance in the dimensions of job performance (except all dimensions of deviant behavior at work, individual task, and team member proficiency). Perseverance explained up to 17.6% of the variance in the dimensions of job performance (except team member total, proficiency, and proactivity; organization member proactivity; interpersonal deviance). Of note, the interpersonal character strengths kindness and social intelligence were relevant predictors for team member adaptivity and proactivity as well as team member total. Additionally, judgment was especially relevant for individual-level performance and its subdimensions individual task proficiency, adaptivity, and proactivity. Further details can be found in Table 8 and Supplementary Table 2.

## Discussion

In the present study, we aimed at investigating the incremental validity of character strengths as predictors of job performance beyond GMA and the Big Five. Furthermore, we aimed at identifying the most important predictors of job performance out of the 24 character strengths, GMA, and the Big Five. In order to achieve a fine-grained overview of the interplay between character strengths and job performance, nine different subdimensions of productive work behavior and two different dimensions of counterproductive work behavior as well as their composites were investigated.

Results of preliminary correlation analyses indicated trustworthiness of data as results from previous research have been replicated. For example, perseverance, teamwork, and leadership were important correlates of job performance (e.g., Harzer and Ruch, 2014; Littman-Ovadia and Lavy, 2016; Harzer et al., 2017). As in previous research, other character strengths were also strongly and meaningfully correlated with specific dimensions of productive and counterproductive work behavior (e.g., Harzer and Ruch, 2014; Littman-Ovadia and Lavy, 2016; Harzer et al., 2017). For example, employees' honesty was positively related to supervisor-rated individual task proficiency, indicating that employees who are able to judge the quality of their work in a realistic way and contribute their share with integrity (e.g., Peterson and Seligman, 2004; Harzer and Ruch, 2014) receive higher ratings in individual task proficiency from their supervisors. Additionally, social intelligence was strongly positively related to team member proficiency, indicating that employees who understand how to fit in in different social situations and what makes other people tick (e.g., Peterson and Seligman, 2004) exhibit higher team member proficiency as rated by their supervisors. Furthermore, employees who had higher scores in fairness and forgiveness received lower scores in overall counterproductive behavior.

Regression analyses indicated incremental validity of character strengths as predictors of job performance beyond GMA and/or the Big Five personality traits (always controlling for employees' sex and age). The research question whether character strengths predict a significant amount of variance in job performance beyond GMA and the Big Five strengths can be answered with a yes. Therefore, in light of these results, character strengths can be considered highly relevant predictors of job performance in terms of productive and counterproductive work behavior above and beyond GMA or the Big Five (as well as both combined). Character strengths showed the numerically strongest incremental validity for team-level performance (especially team member proficiency beyond GMA and team member adaptivity beyond the Big Five) and organization-level performance (especially organization member proficiency beyond GMA and organization member adaptivity beyond the Big Five). This might be due to the larger number of character strengths that positively shape the nature of dyadic or group-related social situations by definition, i.e., the interpersonal strengths, such as kindness and social intelligence, as well as civic strengths, such as teamwork and leadership. Accordingly, those character strengths were among those that showed substantial relative weights in the explorative relative weight analyses. Overall, character strengths concern aspects of personality that are theoretically different from GMAs and the Big Five as argued in the Introduction of the present paper. The results regarding incremental validity indicated that those theoretical differences and empirical differences go hand in hand.

Relative weight analyses were conducted to explore the relative importance of the predictors of job performance in order to answer the research question regarding which predictors among character strengths, GMA, and the Big Five are the most important ones. The results revealed that for each of the dimensions of job performance, at least one character strength explained a numerically higher amount of variance than GMA and the Big Five, except for individual task proactivity, where GMA exhibited the numerically highest amount of explained variance. As in the correlation analyses, perseverance, teamwork, and leadership seemed to be especially relevant for numerous dimensions of job performance. These character strengths seem to be the core of positive work behavior and prevent negative work behavior across occupations; for example, high perseverance helps employees finishing job tasks and not quitting when challenges are faced (e.g., Peterson and Seligman, 2004). Additionally, teamwork supports working well with colleagues, and leadership might help employees understanding, following, and suggesting management decisions on organizational level (e.g., Peterson and Seligman, 2004). The interpersonal character strengths kindness and social intelligence were relevant predictors for team member adaptivity and proactivity as well as team-level performance. This is very meaningful as both character strengths support positive interactions among team members as team members treat each other kindly and understand own and others' emotions and behaviors (e.g., Peterson and Seligman, 2004). Additionally, judgment seemed to be especially relevant for individual-level performance and its subdimensions individual task proficiency, adaptivity, and proactivity. This is very meaningful as behaviors linked to judgment (i.e., thinking things through and examining them from all sides, not jumping to conclusions, being able to change one's mind in light of evidence, weighing all evidence fairly; e.g., Peterson and Seligman, 2004) help employees evaluating their work progress and processes and adapting them if necessary.

### Strengths and Limitations of the Present Study

To the best of our knowledge, the present study is the very first to examine the incremental validity of character strengths as predictors of job performance. Like any other study, the present study has its strengths and weaknesses. The strong points concern (a) its combination of data stemming from different sources (i.e., data from self-reports, an intelligence test, and supervisor ratings), (b) the heterogeneity of the sample, and (c) conservative significance tests applying Bonferroni corrections. Due to the combination of self-reports (character strengths, the Big Five), test data (GMA), and supervisor ratings (job performance), the strong relations between character strengths and job performance cannot be contributed to common method bias (Doty and Glick, 1998). Due to the strategy applied during the recruitment process (i.e., supervisors were recruited, who invited both their poorly and strongly performing team members), the resulting sample was heterogenous with respect to all study variables (except the dimensions of counterproductive work behavior). This led to wide variance in the study variables, and no ceiling effect was observed in the dimensions of productive work behavior, as was the case in Harzer and Ruch (2014). This higher variability in the data in turn led to high reliability coefficients and high correlation coefficients. For example, the correlation between GMA and overall job performance in the present data was similar to the one reported in meta-analyses after correcting for lack of reliability and range restriction (e.g., Schmidt and Hunter, 1998). Finally, conservative significance tests were applied by systematically applying Bonferroni corrections. When identifying relevant correlates of the dimensions of productive and counterproductive work behavior, only character strengths that exhibited a correlation coefficient with a significance level of *p* < 0.0016 were considered in order to control for randomly significant correlations due to the number of significance tests.

Nevertheless, the present study has a number of limitations as well. *Firstly*, results from one relatively small sample of employees from different occupations and sectors were reported. Therefore, studies replicating the results of the present study are needed. Moreover, the results might differ when specific job groups are studied. In the present study, perseverance, teamwork, and leadership were important predictors. However, interpersonal character strengths (love, kindness, social intelligence) are especially relevant in jobs that explicitly involve other people, such as teaching or sales (Peterson and Park, 2006), and could therefore be stronger predictors of job performance in more socially oriented jobs than in the present study. Future research may wish to investigate the role of character strengths and their incremental validity with respect to productive and counterproductive behavior in specific occupations. *Secondly*, as cross-sectional data were reported in the present study, causality could not be inferred, and experimental or longitudinal studies are needed to address this issue. *Thirdly*, in the present study, character strengths and the subdimensions of job performance were on comparable levels of specificity (i.e., narrow concepts). Furthermore, GMA and the Big Five were measured on a higher, more abstract level (i.e., broad concepts), because we wanted to study GMA and the Big Five on the same level of abstraction as reported in well-known meta-analyses (Schmidt and Hunter, 1998; e.g., Salgado and Anderson, 2003). Some studies highlight the role of narrow personality traits (e.g., facets of conscientiousness) and specific aptitudes (e.g., psychomotor abilities) as predictors of job performance (e.g., Schmidt, 2002; Dudley et al., 2006; Grobelny, 2018) as well. Additionally, character strengths and facets of the Big Five overlap (e.g., perseverance as a character strength with achievement thriving and self-discipline as facets of conscientiousness, self-regulation as a character strength with impulsiveness as a facet of neuroticism), although they are not redundant (Noftle et al., 2011; McGrath et al., 2020). Therefore, studies are needed that examine the incremental validity of character strengths beyond specific aptitudes and the facets of the Big Five in order to make sure that all variables share the same level of specificity as narrow traits. However, as the present study combines narrow with broad traits/concepts, its results add information to the bandwidth-fidelity debate (e.g., Cronbach and Gleser, 1957; Salgado et al., 2015). That is, the utilized study design offers the opportunity to get insights into the predictive validity of broad vs. narrow predictors of job performance. Additionally, job performance was operationalized on both the narrow and broad levels. The results of the present study suggest that narrow traits (i.e., character strengths) exhibit incremental validity beyond broad traits (i.e., the Big Five) as predictors of job performance narrowly and broadly construed. Moreover, relative weights of character strengths as narrow traits were numerically higher than those for the Big Five as broad traits. *Fourthly*, a floor effect occurred with respect to counterproductive work behavior (although this was not surprising, as a sample of employees with a reasonably long tenure was studied). The corresponding problem of non-normally distributed data could be solved by transforming the data. Nevertheless, the residuals for regression models with interpersonal deviance as the dependent variable lacked a normal distribution, although they were normally distributed for organizational deviance and overall deviant behavior at work. Therefore, the utilized data analysis methods were not biased for organizational deviance and overall deviant behavior at work, meaning that the results for these variables may be seen as trustworthy. However, the results for interpersonal deviance need to be treated with caution. Additionally, the relations between character strengths and counterproductive work behavior are likely underestimated due to the range restriction. Further studies are needed to obtain better insights here. *Fifthly*, no hypotheses were formulated for the relative weight analyses, which therefore were exploratory in nature. Results from these analyses may now be used for the generation of hypotheses that may be investigated in future studies. *Sixthly*, the structure of the data was nested. Therefore, hierarchical linear modeling might be warranted. However, sample size and cell size did not allow for hierarchical linear modeling. *Seventhly*, each of the dependent variables was investigated independently without taking the intercorrelations among them into account. Future research may wish to systematically investigate the influence of the nested data structure and the correlation among the dependent variables on the results.

### Theoretical and Practical Implications

The results of the present study support theoretical assumptions on the role of character strengths for favorable outcomes at work. Character strengths are defined as positive traits that contribute to a satisfied and successful life (Peterson and Seligman, 2004). The present results support this proposition. Furthermore, the results of the present study show that character strengths exhibit incremental validity as predictors of job performance beyond common predictors, such as GMA and the Big Five. Moreover, relative weights indicated that specific character strengths seem to be important predictors of specific dimensions of job performance. Firstly, this highlights the role of socio-emotional skills, such as character strengths, for understanding performance and success outcomes above and beyond cognitive ability. Secondly, this shows that character strengths are relevant predictors of job performance in addition to broad conceptualizations of personality, such as the Big Five. This underscores the fact that—although the character strengths and the Big Five traits overlap to some degree—they are unique concepts that account for different parts of the variance in outcomes, such as job performance.

The present research showed that individuals with higher scores on specific character strengths receive higher performance ratings from their supervisors. Therefore, it seems meaningful to consider character strengths in personnel selection alongside other common variables. Nevertheless, there are open questions that need to be addressed before applying character strengths (and related assessment measures) as predictors of job candidates' potential future job performance. Research is needed to investigate the direction of causality between character strengths and job performance, as well as possible differences in (a) self-ratings of character strengths and (b) the criterion validity of character strengths when utilized in personnel selection processes (Harzer, 2020). Research shows that applicants' “faking” (i.e., providing more favorable self-descriptions) in personnel selection does not necessarily decrease criterion validity (e.g., Marcus, 2006, 2009). However, this needs to be demonstrated for character strengths as well before they can be utilized to predict future job performance.

## Data Availability Statement

The datasets generated for this study are available on request to the corresponding author.

## Ethics Statement

We strictly followed the “Ethical Principles of Psychologists and Code of Conduct” (American Psychological Association, 2002, 2017) and its adaptation for Germany, with a specific focus on rules for adequate research practice (e.g., rules for informed consent and online research by the German Society of Psychology, 2016). Ethical review and approval was not required for the study on human participants in accordance with the local legislation and institutional requirements. The patients/participants provided their informed consent to participate in this study.

## Author Contributions

CH contributed to the conception and design of the study. NB organized the database. CH and MW performed the statistical analyses and contributed to the final version of the manuscript. CH, NB, and MW wrote the first draft of the manuscript sections and approved the submitted version. All authors contributed to the article and approved the submitted version.

## Conflict of Interest

The authors declare that the research was conducted in the absence of any commercial or financial relationships that could be construed as a potential conflict of interest.

## Footnotes

^1^The R package “personality factors” only allows for hierarchical regression analyses with two steps. We therefore regressed all variables with sex and age and saved the residuals to compute scores for all scales controlling for sex and age.
