# Supplementary material for: Incremental Validity of Character Strengths as Predictors of Job Performance Beyond General Mental Ability and the Big Five
Source: Front Psychol. 2021 Mar 12;12:518369. doi: 10.3389/fpsyg.2021.518369 (PMC7994607; doi:10.3389/fpsyg.2021.518369)
Supplement: Supplementary file 1 [file Table_1.pdf]

SUPPLEMENTARY TABLE 1 | Zero-order Pearson correlations between employees' self-assessed character strengths, GMA, and Big Five (VIA-IS120 scales, CFT 20-R, MRS-25 scales) and supervisor ratings of employees' productive and counterproductive work behavior (WRPS and WDS scales).

Variable	1.	2.	3.	4.	5.	6.	7.	8.	9.	10.	11.	12.	13.	14.	15.	16.	17.	18.	19.	20.	21.	22.	23.	24.
1. Sex																								
2. Age	-.18																							
**VIA-IS120**																								
3. Creativity	.12	.07																						
4. Curiosity	.20	.10	.51																					
5. Judgment	-.14	.33	.30	.33																				
6. Love of learning	.07	.34	.29	.45	.37																			
7. Perspective	-.17	.46	.30	.22	.58	.42																		
8. Bravery	-.15	.31	.41	.33	.69	.35	.58																	
9. Perseverance	-.09	.31	.32	.36	.73	.38	.63	.77																
10. Honesty	.23	.27	.37	.37	.58	.43	.56	.57	.67															
11. Zest	.00	.19	.51	.54	.41	.34	.39	.55	.56	.46														
12. Love	.30	.13	.24	.32	.23	.28	.21	.21	.28	.56	.36													
13. Kindness	.40	.11	.27	.39	.30	.37	.29	.27	.42	.72	.37	.76												
14. Social intelligence	.36	.10	.36	.40	.29	.41	.39	.25	.39	.67	.37	.60	.77											
15. Teamwork	.30	.14	.31	.33	.33	.40	.41	.30	.48	.76	.43	.59	.77	.82										
16. Fairness	.22	.23	.42	.41	.49	.51	.50	.42	.55	.77	.46	.58	.71	.74	.81									
17. Leadership	-.04	.44	.27	.33	.49	.49	.67	.44	.50	.64	.35	.34	.50	.51	.55	.60								
18. Forgiveness	.15	.27	.32	.38	.39	.47	.48	.38	.47	.66	.49	.60	.64	.68	.75	.78	.50							
19. Modesty	.00	.38	.21	.25	.42	.41	.51	.44	.49	.52	.49	.36	.40	.39	.52	.57	.37	.67						
20. Prudence	.16	.31	.23	.32	.72	.29	.43	.50	.57	.53	.30	.32	.36	.34	.40	.48	.39	.48	.50					
21. Self-regulation	.10	.20	.28	.33	.59	.31	.41	.50	.60	.46	.50	.25	.33	.36	.38	.47	.34	.45	.46	.60				
22. Appreciation	.65	-.11	.22	.31	.05	.20	-.03	-.06	.05	.36	.17	.51	.51	.50	.40	.35	.13	.37	.16	.22	.20			
23. Gratitude	.26	.16	.26	.34	.32	.32	.31	.30	.37	.51	.45	.65	.56	.52	.54	.50	.31	.57	.44	.47	.42	.44		
24. Hope	.13	.12	.39	.47	.42	.38	.34	.43	.54	.55	.52	.50	.52	.55	.52	.58	.41	.57	.34	.40	.47	.39	.54	
25. Humor	.04	.10	.34	.50	.31	.38	.35	.40	.46	.44	.49	.35	.55	.37	.44	.45	.45	.44	.31	.27	.31	.13	.31	.43
26. Spirituality	.07	.25	.19	.15	.11	.19	.28	.23	.22	.28	.25	.45	.30	.31	.22	.22	.20	.38	.38	.18	.24	.30	.40	.26
**CFT 20-R**																								
27. GMA	-.10	.08	.13	.17	.36	.10	.32	.33	.43	.24	.32	.21	.14	.16	.23	.27	.26	.22	.18	.17	.33	-.10	.08	.17
**MRS-25**																								
28. Neuroticism	.40	-.21	-.03	-.06	-.36	-.19	-.34	-.36	-.40	-.15	-.19	-.02	.01	-.03	-.06	-.13	-.22	-.18	-.19	-.20	-.31	.17	.00	-.25
29. Extraversion	.13	-.07	.14	.06	-.03	.14	.14	.06	.18	.26	.30	.33	.40	.51	.49	.34	.31	.33	.15	-.07	.13	.26	.26	.31
30. Culture	.23	-.19	.47	.25	.07	.19	.09	.18	.19	.21	.25	.30	.32	.39	.31	.27	.14	.26	.08	.01	.19	.32	.24	.30
31. Agreeableness	.31	.08	.16	.17	.15	.33	.19	.11	.28	.41	.28	.43	.50	.49	.60	.56	.25	.57	.50	.30	.34	.37	.44	.29
32. Conscientiousness	.05	.21	.22	.12	.42	.32	.40	.33	.47	.44	.23	.16	.25	.33	.37	.46	.40	.39	.32	.32	.42	.21	.18	.31
**WRPS**																								
33. Overall job performance	-.04	.29	.37	.38	.56	.50	.54	.52	.66	.61	.48	.32	.48	.47	.62	.60	.70	.52	.39	.39	.44	.05	.34	.45
34. Individual-level perf.	-.07	.28	.40	.38	.60	.46	.50	.57	.71	.54	.52	.27	.39	.37	.50	.53	.57	.44	.38	.41	.48	.00	.33	.43
35. Individual task prof.	-.09	.28	.29	.25	.59	.35	.50	.53	.75	.56	.50	.27	.40	.41	.53	.52	.50	.42	.36	.37	.50	-.01	.36	.42
36. Individual task adapt.	-.03	.19	.44	.41	.55	.41	.45	.56	.63	.49	.49	.25	.37	.31	.43	.48	.52	.41	.36	.40	.43	.03	.28	.36
37. Individual task proac.	-.06	.28	.34	.36	.47	.45	.39	.41	.50	.40	.39	.20	.26	.27	.37	.40	.49	.34	.29	.32	.35	-.01	.25	.35
38. Team-level performance	.05	.22	.34	.34	.47	.48	.48	.39	.55	.61	.42	.41	.56	.59	.73	.64	.63	.57	.39	.34	.40	.15	.40	.44
39. Team member prof.	.14	.10	.21	.19	.28	.33	.36	.20	.42	.57	.35	.42	.58	.64	.80	.61	.45	.56	.37	.24	.36	.22	.41	.36
40. Team member adapt.	.02	.19	.40	.34	.49	.45	.46	.44	.56	.60	.41	.37	.53	.53	.65	.59	.63	.50	.34	.36	.36	.14	.38	.41
41. Team member proac.	-.04	.28	.30	.36	.46	.49	.45	.38	.48	.45	.35	.28	.37	.39	.49	.49	.58	.45	.32	.31	.32	.04	.27	.38
42. Organ.-level performance	-.10	.31	.30	.34	.50	.45	.52	.49	.59	.56	.41	.23	.40	.37	.50	.53	.75	.44	.33	.33	.35	.00	.23	.39
43. Organ. member prof.	.01	.29	.30	.31	.49	.45	.52	.48	.62	.60	.50	.32	.47	.48	.60	.59	.67	.54	.40	.39	.43	.12	.35	.49
44. Organ. member adapt.	-.12	.22	.31	.35	.48	.37	.44	.46	.54	.50	.38	.16	.35	.28	.41	.44	.66	.36	.29	.30	.29	-.03	.19	.30
45. Organ. member proac.	-.15	.31	.19	.24	.39	.39	.43	.37	.44	.42	.24	.15	.27	.24	.34	.39	.69	.30	.21	.19	.22	-.08	.09	.26
**WDS**																								
46. Overall deviant behavior at work	.00	-.19	-.25	-.16	-.36	-.35	-.47	-.33	-.50	-.51	-.40	-.39	-.47	-.51	-.62	-.57	-.47	-.56	-.33	-.22	-.42	-.14	-.38	-.41
47. Interpersonal deviance	-.16	-.02	-.04	-.02	-.06	-.22	-.17	-.03	-.15	-.28	-.12	-.28	-.31	-.39	-.41	-.37	-.19	-.39	-.13	-.07	-.21	-.20	-.26	-.23
48. Organizational deviance	.12	-.28	-.34	-.20	-.48	-.32	-.52	-.48	-.62	-.51	-.48	-.31	-.40	-.40	-.53	-.53	-.50	-.47	-.38	-.26	-.43	-.01	-.31	-.40

(SUPPLEMENTARY TABLE 1 continues)

SUPPLEMENTARY TABLE 1 (continued) | Zero-order Pearson correlations between employees' self-assessed character strengths, GMA, and Big Five (VIA-IS120 scales, CFT 20-R, MRS-25 scales) and supervisor ratings of employees' productive and counterproductive work behavior (WRPS and WDS scales).

Variable	25.	26.	27.	28.	29.	30.	31.	32.	33.	34.	35.	36.	37.	38.	39.	40.	41.	42.	43.	44.	45.	46.	47.
1. Sex																							
2. Age																							
**VIA-IS120**																							
3. Creativity																							
4. Curiosity																							
5. Judgment																							
6. Love of learning																							
7. Perspective																							
8. Bravery																							
9. Perseverance																							
10. Honesty																							
11. Zest																							
12. Love																							
13. Kindness																							
14. Social intelligence																							
15. Teamwork																							
16. Fairness																							
17. Leadership																							
18. Forgiveness																							
19. Modesty																							
20. Prudence																							
21. Self-regulation																							
22. Appreciation																							
23. Gratitude																							
24. Hope																							
25. Humor																							
26. Spirituality	.17																						
**CFT 20-R**																							
27. GMA	.07	.19																					
**MRS-25**																							
28. Neuroticism	-.12	-.04	-.13																				
29. Extraversion	.23	.18	.08	-.10																			
30. Culture	.23	.14	.13	.03	.47																		
31. Agreeableness	.24	.22	.01	-.06	.36	.24																	
32. Conscientiousness	.23	.11	.08	-.42	.21	.19	.43																
**WRPS**																							
33. Overall job performance	.49	.16	.39	-.29	.27	.24	.33	.43															
34. Individual-level perf.	.42	.16	.44	-.31	.19	.25	.28	.39	.94														
35. Individual task prof.	.32	.16	.37	-.32	.30	.22	.32	.44	.82	.87													
36. Individual task adapt.	.48	.10	.41	-.24	.10	.28	.23	.35	.85	.90	.68												
37. Individual task proac.	.32	.18	.40	-.27	.11	.16	.18	.26	.84	.89	.64	.71											
38. Team-level performance	.44	.17	.37	-.24	.33	.26	.39	.40	.93	.82	.73	.71	.73										
39. Team member prof.	.31	.16	.33	-.15	.45	.28	.46	.36	.68	.56	.63	.44	.43	.85									
40. Team member adapt.	.46	.13	.32	-.18	.25	.26	.33	.39	.88	.79	.69	.79	.63	.91	.71								
41. Team member proac.	.39	.17	.32	-.28	.18	.15	.24	.31	.87	.79	.60	.65	.85	.87	.54	.71							
42. Organ.-level performance	.50	.11	.28	-.27	.23	.17	.27	.41	.93	.82	.68	.77	.72	.78	.50	.77	.77						
43. Organ. member prof.	.45	.14	.22	-.23	.30	.20	.34	.41	.82	.75	.74	.64	.61	.72	.59	.69	.62	.84					
44. Organ. member adapt.	.46	.08	.26	-.19	.15	.16	.23	.37	.84	.74	.57	.78	.61	.69	.41	.75	.65	.92	.65				
45. Organ. member proac.	.41	.08	.27	-.29	.18	.09	.18	.33	.83	.71	.53	.63	.71	.69	.37	.64	.79	.91	.62	.80			
**WDS**																							
46. Overall deviant behavior at work	-.32	-.18	-.27	.31	-.43	-.32	-.38	-.39	-.68	-.64	-.72	-.49	-.48	-.72	-.72	-.63	-.54	-.55	-.65	-.41	-.44		
47. Interpersonal deviance	-.08	-.11	-.04	.10	-.27	-.21	-.34	-.16	-.31	-.23	-.30	-.14	-.17	-.42	-.54	-.33	-.24	-.23	-.38	-.12	-.13	.72	
48. Organizational deviance	-.37	-.14	-.35	.36	-.38	-.29	-.26	-.42	-.72	-.74	-.81	-.61	-.55	-.68	-.58	-.63	-.57	-.61	-.63	-.49	-.52	.84	.25

Note. N = 169. *WRPS* = Work Role Performance Scale (Griffin et al., 2007): *prof.* = proficiency, *adapt.* = adaptivity, *proact.* = proactivity. *WDS* = Workplace Deviance Scale (Bennett & Robinson, 2000). *VIA-IS120* = Values in Action Inventory of Strengths (Littman-Ovadia, 2015). *CFT 20-R* = Revised Culture Fair Intelligence Test Scale 2 (Weiß, 2006). *GMA* = General mental ability. *MRS-25* = Minimal Redundancy Scales (Ostendorf, 1990). Significance cut-off: Correlation coefficients ≥ |.25| were significant at $p < .0016$.
