# Supplementary material for: Incremental Validity of Character Strengths as Predictors of Job Performance Beyond General Mental Ability and the Big Five
Source: Front Psychol. 2021 Mar 12;12:518369. doi: 10.3389/fpsyg.2021.518369 (PMC7994607; doi:10.3389/fpsyg.2021.518369)
Supplement: Supplementary file 2 [file Table_2.pdf]

SUPPLEMENTARY TABLE 2 | Relative weights (RW) and percentages of explained criterion variance (%) for all character strengths, GMA, and Big Five (VIA-IS120 scales, CFT 20-R, MRS-25 scales) for productive and counterproductive work behavior (WRPS and WDS scales).

	**WRPS**																		**WDS**			
	**Individual task**						**Team member**						**Organization member**									
	**Prof**		**Adapt**		**Proact**		**Prof**		**Adapt**		**Proact**		**Prof**		**Adapt**		**Proact**		**Int**		**Org**	
**Predictor**	RW	%	RW	%	RW	%	RW	%	RW	%	RW	%	RW	%	RW	%	RW	%	RW	%	RW	%
**VIA-IS120**																						
Creativity	.009	1.3	.043*	7.1	.028*	6.0	.003	0.5	.033*	5.1	.015	3.0	.007	1.1	.015	2.6	.004	0.7	.008	2.5	.022*	3.7
Curiosity	.006	0.8	.020*	3.2	.021	4.5	.003	0.5	.008	1.3	.018	3.7	.007	1.1	.026	4.4	.010	1.8	.012	3.5	.005	0.9
Judgment	.057*	8.2	.033*	5.5	.032*	6.9	.008	1.2	.025*	3.9	.029*	5.8	.021	3.4	.027	4.5	.016	2.9	.004	1.1	.026*	4.5
Love of learning	.008	1.1	.023*	3.8	.052*	11.0	.013	1.7	.029*	4.5	.051*	10.4	.022	3.5	.016	2.8	.026*	4.6	.013	3.7	.004	0.7
Perspective	.018*	2.6	.014*	2.2	.008	1.7	.013*	1.8	.016*	2.4	.014*	2.8	.022	3.6	.018	3.1	.019*	3.4	.010	3.1	.025*	4.2
Bravery	.030*	4.4	.037*	6.0	.010	2.2	.008	1.2	.015*	2.2	.007	1.5	.018	3.0	.019	3.2	.010	1.8	.006	1.7	.018*	3.0
Perseverance	.122*	17.6	.057*	9.4	.029*	6.1	.019*	2.7	.032*	5.0	.020*	4.1	.054*	8.7	.036*	6.1	.018	3.1	.006	1.7	.048*	8.2
Honesty	.032*	4.6	.018*	2.9	.012	2.5	.040*	5.6	.032*	4.9	.014	2.9	.028	4.5	.035*	5.9	.029*	5.2	.010	3.0	.021*	3.6
Zest	.034*	4.9	.027*	4.5	.013	2.8	.011*	1.6	.010	1.6	.007	1.4	.036*	5.8	.014	2.3	.004	0.7	.005	1.4	.025*	4.2
Love	.007	1.0	.005	0.8	.003	0.7	.016*	2.2	.012*	1.8	.007	1.3	.007	1.1	.006	0.9	.003	0.6	.010	2.9	.012	2.1
Kindness	.021*	3.0	.017*	2.7	.006	1.4	.045*	6.3	.035*	5.3	.014	2.8	.020	3.2	.023	3.8	.014	2.5	.009	2.7	.024*	4.0
Social intelligence	.018*	2.6	.008	1.3	.007	1.5	.065*	9.0	.034*	5.2	.016*	3.3	.020	3.3	.012	2.0	.010	1.8	.022	6.4	.017*	2.8
Teamwork	.046*	6.6	.020*	3.3	.021	4.5	.157*	21.8	.082*	12.5	.042*	8.6	.050*	8.1	.026	4.4	.021*	3.8	.029	8.4	.049*	8.3
Fairness	.021*	3.1	.014*	2.3	.012	2.5	.041*	5.7	.026*	4.0	.019*	3.8	.026	4.2	.018	2.9	.018*	3.2	.027	7.9	.025*	4.2
Leadership	.022*	3.2	.053*	8.7	.047*	10.1	.024*	3.4	.083*	12.7	.072*	14.7	.105*	16.9	.155*	26.1	.193*	34.4	.006	1.7	.021*	3.6
Forgiveness	.011	1.5	.012	1.9	.006	1.4	.035*	4.8	.020*	3.0	.017*	3.5	.026	4.2	.010	1.7	.007	1.3	.041	12.0	.018*	3.0
Modesty	.008	1.1	.008	1.2	.003	0.6	.013*	1.8	.006	1.0	.004	0.9	.008	1.2	.004	0.6	.004	0.7	.013	3.9	.009	1.4
Prudence	.016*	2.4	.015*	2.4	.008	1.7	.006	0.8	.009	1.3	.007	1.4	.009	1.5	.009	1.4	.007	1.2	.009	2.6	.012*	2.0
Self-regulation	.034*	4.9	.014*	2.3	.009	1.9	.013*	1.9	.007	1.1	.005	1.1	.017	2.7	.005	0.9	.003	0.6	.014	4.2	.024*	4.1
Appreciation	.012	1.8	.004	0.7	.009	2.0	.004	0.5	.003	0.5	.007	1.5	.003	0.5	.007	1.2	.006	1.1	.002	0.6	.003	0.6
Gratitude	.019*	2.7	.007	1.1	.007	1.5	.024*	3.3	.016*	2.5	.005	1.0	.007	1.1	.003	0.5	.004	0.7	.009	2.8	.012	1.9
Hope	.016*	2.3	.008	1.3	.011	2.3	.009	1.2	.009	1.4	.012	2.5	.025	4.0	.007	1.2	.007	1.3	.006	1.8	.010	1.8
Humor	.008	1.2	.038*	6.2	.012	2.5	.009	1.3	.027*	4.2	.020*	4.0	.022	3.6	.037*	6.3	.051*	9.2	.006	1.9	.013	2.1
Spirituality	.002	0.3	.004	0.6	.005	1.1	.002	0.3	.002	0.4	.002	0.4	.002	0.4	.001	0.2	.001	0.1	.002	0.6	.003	0.5
**CFT 20-R**																						
GMA	.023*	3.3	.051*	8.4	.060*	12.8	.038*	5.2	.024*	3.7	.025*	5.1	.006	0.9	.010	1.7	.022	3.9	.004	1.1	.020*	3.5
**MRS-25**																						
Neuroticism	.012	1.7	.007	1.1	.018	3.8	.009	1.2	.003	0.5	.018	3.7	.004	0.7	.003	0.5	.014	2.6	.015	4.5	.022*	3.8
Extraversion	.021*	3.1	.009	1.5	.003	0.7	.037*	5.1	.009	1.4	.006	1.1	.013	2.2	.007	1.1	.009	1.7	.008	2.3	.042*	7.0
Culture	.012	1.7	.026*	4.2	.007	1.5	.009	1.3	.011	1.6	.004	0.8	.007	1.1	.007	1.2	.004	0.7	.012	3.5	.027*	4.6
Agreeableness	.017*	2.5	.006	0.9	.004	0.8	.027*	3.7	.012*	1.9	.006	1.3	.011	1.7	.012	2.0	.010	1.8	.020	5.8	.007	1.2
Conscientiousness	.031*	4.4	.015*	2.4	.005	1.0	.018*	2.5	.021*	3.2	.008	1.6	.018	2.9	.025	4.3	.014	2.5	.003	1.0	.026*	4.4
**R^2**	.692	100	.612	100	.469	100	.719	100	.651	100	.490	100	.623	100	.595	100	.561	100	.341	100	.591	100

Note. $N = 169$. All data were corrected for effects of sex and age before being entered into the regression analyses. RW = Raw relative weight (within rounding error, raw weights sum up to R^2), % = Relative weight rescaled to as a percentage of predicted variance in the criterion attributed to each predictor (within rounding error, rescaled weights sum to 100). *WRPS* = Work Role Performance Scale (Griffin et al., 2007): *Prof* = Proficiency, *Adapt* = Adaptivity, *Proact* = Proactivity. *WDS* = Workplace Deviance Scale (Bennett & Robinson, 2000): *Int* = Interpersonal deviance, *Org* = Organizational deviance. *VIA-IS120* = Values in Action Inventory of Strengths (Littman-Ovadia, 2015). *CFT 20-R* = Revised Culture Fair Intelligence Test Scale 2 (Weiß, 2006). *GMA* = General mental ability. *MRS-25* = Minimal Redundancy Scales (Ostendorf, 1990).
* 95% percent confidence interval did not include zero ($p < .05$).
